# A systematic review and meta-analysis of *Lactobacillus acidophilus and Lactobacillus bulgaricus* for the treatment of diarrhea

**DOI:** 10.3389/fgstr.2022.983075

**Published:** 2022-11-09

**Authors:** Anthony Carona, David Jacobson, Charles F. Hildebolt, Waqar Qureshi, Kevin C. Rowland

**Affiliations:** ^1^ Department of Biomedical Sciences, Tilman J. Fertitta Family College of Medicine, University of Houston, Houston, United States; ^2^ Department of Radiology, Washington University School of Medicine, St. Louis, United States; ^3^ Division of Gastroenterology, Baylor College of Medicine, Houston, United States

**Keywords:** antibiotic associated diarrhea, travelers’ diarrhea, probiotics, lactinex, lactobacillus acidophilus, Lactobacillus bulgaricus

## Abstract

**Background and aims:**

Probiotics are widely used and prescribed to address a host of health issues. Despite evidence that different probiotic bacteria have differing therapeutic mechanisms of action, many probiotics are prescribed indiscriminately, with little research to support the use of specific formulations for a given ailment. Further investigation is required to assess the efficacy of one commonly prescribed probiotic formulation *Lactobacillus acidophilus* and *Lactobacillus bulgaricus* (*helveticus*) – for the treatment of diarrhea. This review seeks to assess whether administration of probiotics composed of *L.acidophilus* and *L. bulgaricus (helveticus*) are more effective than placebo in reducing symptoms of diarrhea.

**Methods:**

A systematic search of randomized placebo-controlled trials evaluating the effectiveness of combination *L. acidophilus* and *L. bulgaricus* in the treatment of diarrhea by any cause was conducted and captured all available studies (n = 2411). After application of exclusion criteria, four studies were identified as suitable for inclusion. Separate meta-analyses were conducted for the proportion of cases with diarrhea in the placebo group and the treatment group. To assess differences in proportions between the placebo and treatment groups, a generalized linear model assessment was performed.

**Results:**

Analyses revealed the overall proportion of cases with diarrhea in the treatment group, 36 participants who had diarrhea out of 91 total, was only 3.5% lower than the overall proportion in the placebo group, 44 participants who had diarrhea out of 105 total.(P = 0.508), with our considering that the 3.5 lower percentage to be of little or no clinical importance.

**Conclusion:**

Existing literature suggests little or no clinical benefit of a *L. acidophilus* and *L. bulgaricus* probiotic formulation for the treatment of diarrhea, highlighting the need for more research or re-evaluation of its widespread use.

## 1 Introduction

Probiotic usage has long been sought to treat a wide range of ailments including Celiac’s disease, diarrhea, bacterial infections, autism, vaginal dysbiosis, and a host of other medical conditions (1–5). A growing body of data is shedding light on probiotic efficacy in particular therapeutic circumstances, especially symptomatic relief of gastrointestinal diseases (6–8). Progress toward elucidating the mechanisms of action of probiotic organisms is also being made, though many such mechanisms appear to be strain specific (9–11).

One commonly prescribed probiotic combination consists of *Lactobacillus acidophilus* and *Lactobacillus bulgaricus (helveticus)* packaged as 500 mg tablets or a 1 g packet of granules. Even in the absence of any recommendations for its use, this specific formulation was found to be the second most stocked probiotic in academic hospitals nationwide (12). Strain specificity may be problematic given current practice – where many consumers utilize probiotics without knowledge of the specific organisms they contain. In fact, many commercially available probiotics do not even name the specific microorganisms present in the product (7). To this point, little evidence exists to support strain specific recommendations for relief of a given illness (1, 4, 7, 13). Absence of research efficacy impairs physicians’ abilities to optimize therapeutic use of probiotics. Instead, formulations are recommended indiscriminately, as if various bacteria were generically equivalent, despite evidence to the contrary (7, 9–11, 13).

Recognizing the need to assess efficacy in a therapeutically specific manner, this review seeks to evaluate the effectiveness of the *L. acidophilus* and *L. bulgaricus* probiotic formulation in the treatment of diarrhea, regardless of cause. Due to the scarcity of available studies, a systematic review and meta-analysis of published studies was performed to determine the effect of a *L. acidophilus* and *L. bulgaricus* probiotic in the treatment of diarrhea. The individual causes of diarrhea that were studied against *L. acidophilus* and *L. bulgaricus* were: antibiotic associated diarrhea from amoxicillin, antibiotic associated diarrhea from ampicillin, diarrhea due to *Escherichia coli*, and travelers’ diarrhea.

The *L. acidophilus* and *L. bulgaricus* formulation under review forms part of a growing probiotic industry worth 54.21 billion USD and marketed toward nearly two thirds of all Americans burdened by gastrointestinal symptoms (14, 15). Given the persistent and widespread use of this probiotic, it is, therefore, of great clinical importance to evaluate its effectiveness.

## 2 Methods

### 2.1 Design

The systematic review of the literature was performed with a blinded consensus of the available studies *via* Rayyan QCRI (16). In areas of disagreement, all three reviewers would convene to make a unanimous decision upon full text review.

### 2.2 Systematic literature search

All available trials evaluating the effectiveness of a combination of *L. acidophilus* and *L. bulgaricus* (*Helveticus*) (17) by any name, including trade names Lactinex and Floranex, in the treatment of diarrhea by any cause were identified by a July 16^th^, 2021 literature review–based on Appraisal of Guidelines, Research and Evaluation [AGREE] II criteria (18). The search terms (**Table 1**) were used to identify any relevant articles that were available in Pubmed, Cochrane, CINAHL, Embase, Web of Science, and Clinicaltrials.gov. To ensure the most current information was included, an updated database search was completed on March 14, 2022, and yielded 98 additional articles, none of which met the inclusion criteria.

**Table 1:** Article review inclusion and exclusion criteria.

	Inclusion criteria	Exclusion criteria
Population	Any	
Interventions	*Lactobacillus acidophilus* & *Lactobacillus bulgaricus*	Any additional probiotic strain that is not *L. acidophilus* and *L. bulgaricus*
Outcomes	Diarrhea	
Study design	Randomized Controlled Trials Placebo‐controlled trials Studies with a clear sample size calculation	Meta‐analysis Systematic review
Date restrictions	None	
Language restrictions	Full text - English language	
Country	Not restricted by country	

#### 2.2.1 Search criteria

Due to the diverse selection of indices, the search criteria was modified for each index.

Pubmed: [(“Lactobacillus acidophilus”[Mesh] OR lactobacillus acidophilus[tiab]) AND ((Lactobacillus bulgaricus[tiab] OR Lactobacillus helveticus[tiab] OR “Lactobacillus delbrueckii”[Mesh]) OR (Lactinex[tiab] OR Floranex[tiab])]

Cochrane: (“Lactobacillus acidophilus”) AND [(Lactobacillus bulgaricus OR Lactobacillus helveticus OR “Lactobacillus delbrueckii”) OR (Lactinex OR Floranex)]

CINAHL: (“Lactobacillus acidophilus”) AND [(Lactobacillus bulgaricus OR Lactobacillus helveticus OR “Lactobacillus delbrueckii”) OR (Lactinex OR Floranex)]

Embase: (Lactobacillus acidophilus.mp.) AND (Lactobacillus bulgaricus.mp. OR Lactobacillus helveticus.mp. OR Lactobacillus delbrueckii.mp.) OR (Lactinex.tw. OR Floranex.tw.)

Web of Science: (“Lactobacillus acidophilus”) AND [(Lactobacillus bulgaricus OR Lactobacillus helveticus OR “Lactobacillus delbrueckii”) OR (Lactinex OR Floranex)]

Clinicaltrials.gov: Keyword searches were used due to index limitations.

Keywords: Lactobacillus acidophilus and Lactobacillus bulgaricus &

Lactobacillus acidophilus and Lactobacillus helveticus & lactinex floranex

The search criteria was intended to maximize the available articles and ensure that a relevant article was not missed due to synonyms, for this reason, outcomes were not included in the search criteria.

#### 2.2.2 Inclusion and exclusion criteria

As seen in **Table 1**, the criteria used to evaluate appropriate articles is as follows. To be included, the intervention needed to be a combination of *L. acidophilus* and *L. Bbulgaricus* with an outcome of diarrhea. Additionally, the study needed to have design of randomly controlled or placebo controlled trials with a clear sample size. Finally, the studies needed to have a full text available in English. The exclusion criteria were any probiotic supplement that was not exclusively *L. acidophilus and L. bulgaricus* or a study design of meta-analysis or systematic review.

### 2.3 Citation screening and full‐text review

The 2009 Preferred Reporting Items for Systematic Reviews and Meta‐Analyses (PRISMA) (19) guidelines were followed while titles and abstracts of all identified articles were screened for inclusion criteria (**Table 1**). This review was not registered prior to commencement. Upon selection of articles that may be appropriate, a review of the full text was performed to confirm article eligibility.

### 2.4 Data items collected and quality assessment

The same data items were collected and tabulated as in the original systematic review, including patient demographics, sample size, strain of probiotic, setting, primary and secondary endpoints, and results. Of note, the term “probiotics” has been used throughout to refer to products that contain probiotics, regardless of whether these are single or multiple strains. The additional step of a quality assessment was performed for each publication (in both the original and the updated review) using a modified version of the Critical Appraisal Skills Programme (CASP) Checklist for Randomised Controlled Trials (20), as recommended by the National Institute for Health and Care Excellence (21).

### 2.5 Consensus method

Consensus was obtained by the reviewers utilizing a third reviewer. This process allowed for any disagreements between inclusion and exclusion that arose during the blinded selection to be discussed with both initial reviewers along with the third reviewer to work towards consensus. Inclusion and exclusion were based only on the pre-defined inclusion and exclusion criteria. All disagreements were able to be managed this way. However, had this not been successful, a vote would have been taken, and the majority would have decided.

### 2.6 Statistical analyses

**Figure 1:**
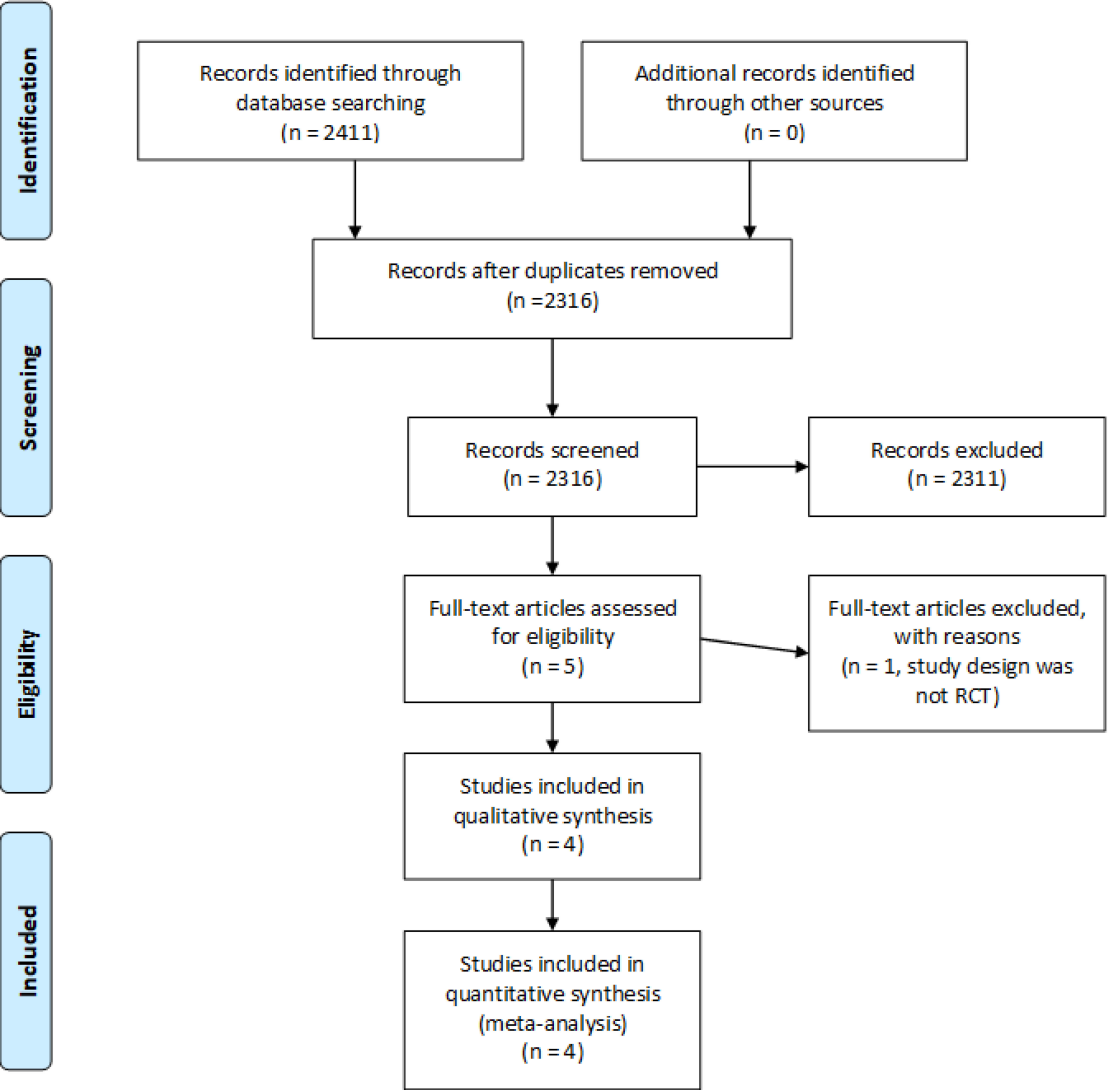
Flow diagram identification, screening and inclusion of studies.

**Figure 2:**
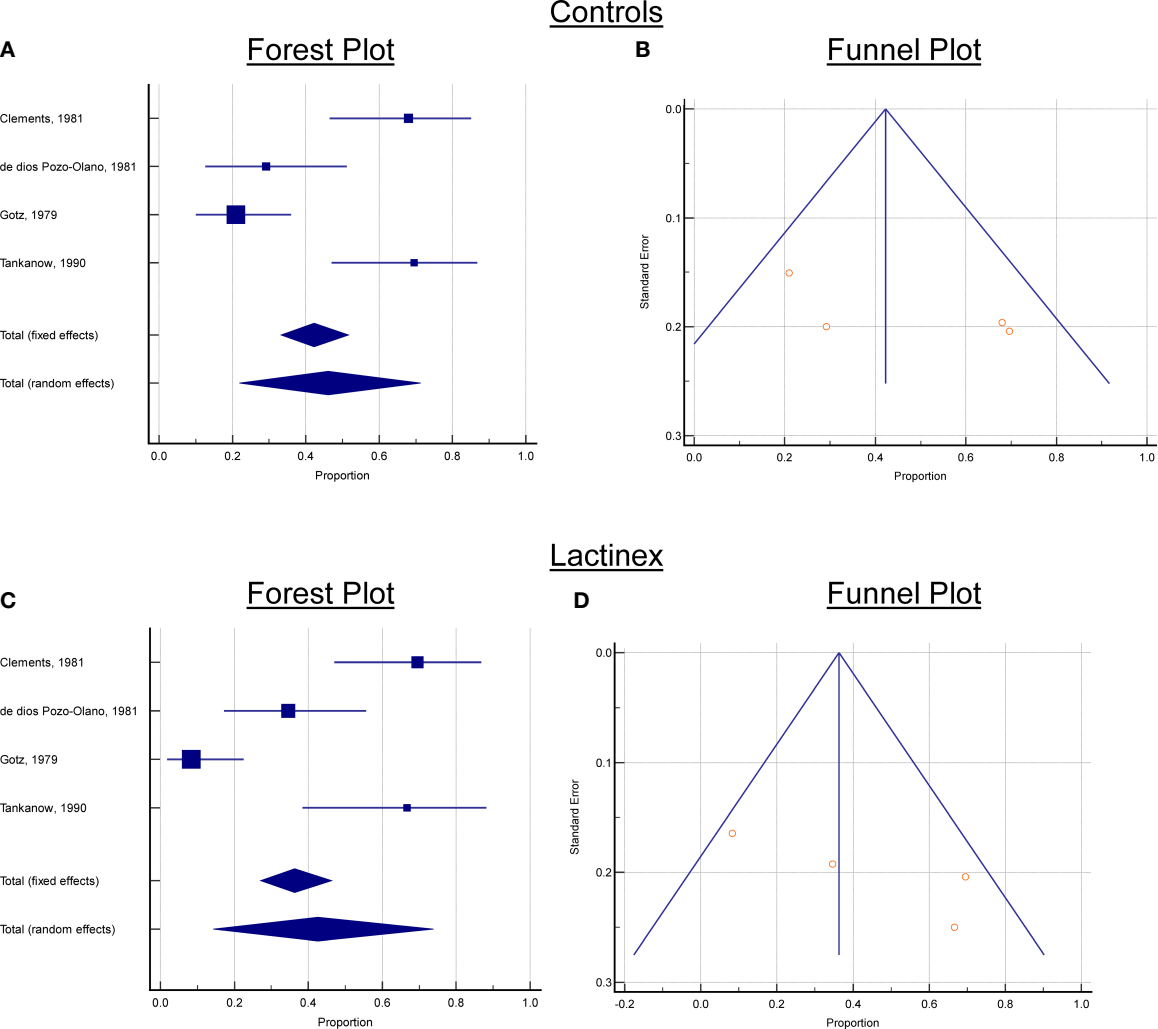
Forest and Funnels plots. **(A)** Controls, Forest plot, **(B)** Controls, Funnel Plot, **(C)** Lactinex, Forest plot, **(D)** Lactinex, Funnel Plot. Each Forest plot contains the overall standardized mean differences and 95% confidence intervals for each article and the overall effects (under the fixed and random effects model). If 0 is not within the 95% confidence interval, the standardized mean difference (SMD) is significant at the 5% level. For each Funnel plot,the treatment effect is plotted on the horizontal axis, and the standard error is on the vertical axis. The vertical line represents the summary estimate derived using fixed/random-effect meta-analysis. The two diagonal lines represent(pseudo) 95% confidence limits (effect ± 1.96 SE) around the summary effect for each standard error on the the vertical axis. These show the expected distribution of articles in the absence of heterogeneity or of selection bias. In the absence of heterogeneity, 95% of the articles should lie within the funnel defined by these diagonal lines.

**Figure 3:**
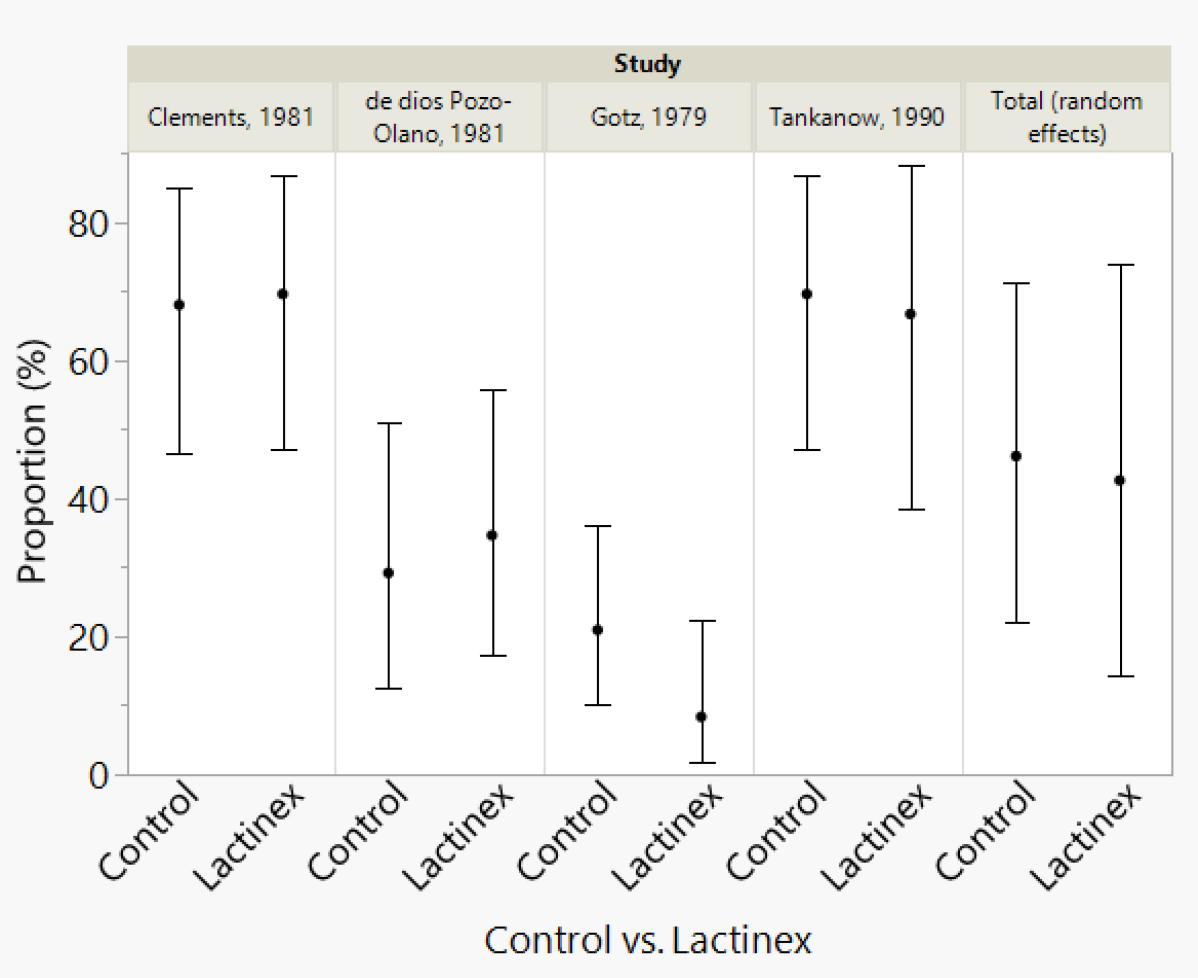
Meta-analysis results for proportions of diarrhea for each study and the total random effects proportions for control and Lactinex. The vertical error bars represent the 95% confidence intervals.

Based upon the systematic review, for the selected articles, the following data were collected from each article: (1) the number of placebo cases who developed diarrhea, (2) the total number of placebo cases, (3) the number of probiotic treated cases who developed diarrhea, (4) the total number of probiotic treated cases. With these data, two meta-analyses of the proportion of cases with diarrhea were performed—one meta-analysis for cplacebo and one meta-analysis for *L. acidophilus* and *L. bulgaricus* treatment. Assessments of publication bias were performed with Egger’s test and Begg’s test. To assist in interpreting meta-analysis results, a forest plot and funnel plot were created for each meta-analysis (**Figure 1**). **Figure 2** was also created to make it easier for readers to assess the meta-analysis results for proportions of diarrhea for each study and the total random effects proportions for cplacebo and treatment.An additional goal of our assessments was to determine whether there was a significant difference between cplacebo and probiotic groups for the overall proportions of cases who developed diarrhea, and for this assessment, we wanted to include the effect of the four articles. To assess differences in proportions between placebo and probiotic, we used a generalized linear model and created a prediction profiler to illustrate the variation among articles compared with the variation between treatments (**Figure 3**).

In a recently published article, it was recommended that the clinical importance of findings needs to be stressed, not merely their statistical significance (22). With this recommendation in mind, we thought that rather than readers trying to assess the clinical importance of the difference in the overall proportions for placebo and probiotic treatment based upon our generalized linear model analysis, it would be relatively easy for them to assess the clinical importance of the difference between the overall proportions for cplacebo and for probiotic treatment that were calculated with meta-analyses. Because of this, we performed a power analysis (sample-size calculation) for testing the difference between two independent proportions (as determined with the meta-analyses) for cplacebo and probiotic treatment. For the analyses that were performed for our study, the alpha level was set at 0.05. Statistical analyses were performed with JMP Pro Statistical Software Release 16.2.0 (SAS Institute, Inc., Cary, NC), MedCalc Statistical Software version 20.104 (MedCalc Software Ltd, Ostend, Belgium; https://www.medcalc.org; 2022), and Power and Precision Release 4.1 (Biostat, Inc., Englewood, NJ).

Details of our statistical analyses are presented in **Supplementary Materials**.

## 3 Results

Our search strategy yielded a total of four articles that met inclusion criteria with three distinct diarrhea outcomes: antibiotic associated diarrhea (Gotz, 1979 and Tankankow, 1990); traveler’s diarrhea (Pozo-Olano, 1978); and diarrhea due to enterotoxigenic *E. coli* (Clements, 1981) (**Table 2**; 23–26).

**Table 2:** Characteristics of included studies including sample sizes, diarrhea cause, treatment modality and summary of findings.

Study	Population Size	Cause of Diarrhea	Strain of Probiotic used	Dose and Dosing schedule	Results / Outcome
Tankanow, 1990 (23)	38 children between the ages of 5 months to 6 years Lactinex, n =15 Placebo, n = 23	Amoxicillin induced diarrhea	Lactinex - *Lactobacillus acidophilus* and *Lactobacillus bulgaricus*	1g Packet of Lactinex – 4 times per day vs Placebo	No change in incidence of diarrhea
Pozo-Olano, 1978 (24)	50 healthy adults, age 18 or older Lactinex, n =26 Placebo, n = 24	Travelers’ Diarrhea	Lactinex - *Lactobacillus acidophilus* and *Lactobacillus bulgaricus*	4 Tablets – 3 Times per day with meals	No statistically significant difference between placebo and treatment
Clements, 1981 (25)	48 healthy adults, age 18-35 Lactinex, n =23 Placebo, n = 25	Diarrhea due to *E. coli*	Lactinex - *Lactobacillus acidophilus* and *Lactobacillus bulgaricus*	1g Packet of Lactinex – 4 times per day vs Placebo	No difference between incidence between placebo and treatment, treatment group had slightly higher severity of disease.
Gotz, 1979 (26)	79 adults who required Ampicillin treatment Lactinex, n =36 Placebo, n = 43	Ampicillin associated diarrhea	Lactinex - *Lactobacillus acidophilus* and *Lactobacillus bulgaricus*	1g Packet of Lactinex – 4 times per day vs Placebo	No statistically relevant difference between treatment and placebo*

*The authors of this study hypothesized that the treatment may be effective after excluding 6 patients from the treatment group, for further information see Gotz et. al, 1979.

### 3.1 Meta-analysis of placebo

For the meta-analysis of placebo, there was considerable heterogeneity (P < 0.0001), and this indicated that the proportion determined with random-effects model should be used to interpret results. This proportion was 46.1% (21.9%─71.3, 95% confidence interval). Neither Begg’s test (P = 0.1742) nor Egger’s test (P = 0.2403) indicated publication bias. The forest and funnels plots for placebo are presented in **Figure 2**.

### 3.2 Meta-analysis of treatment

**Figure 4:**
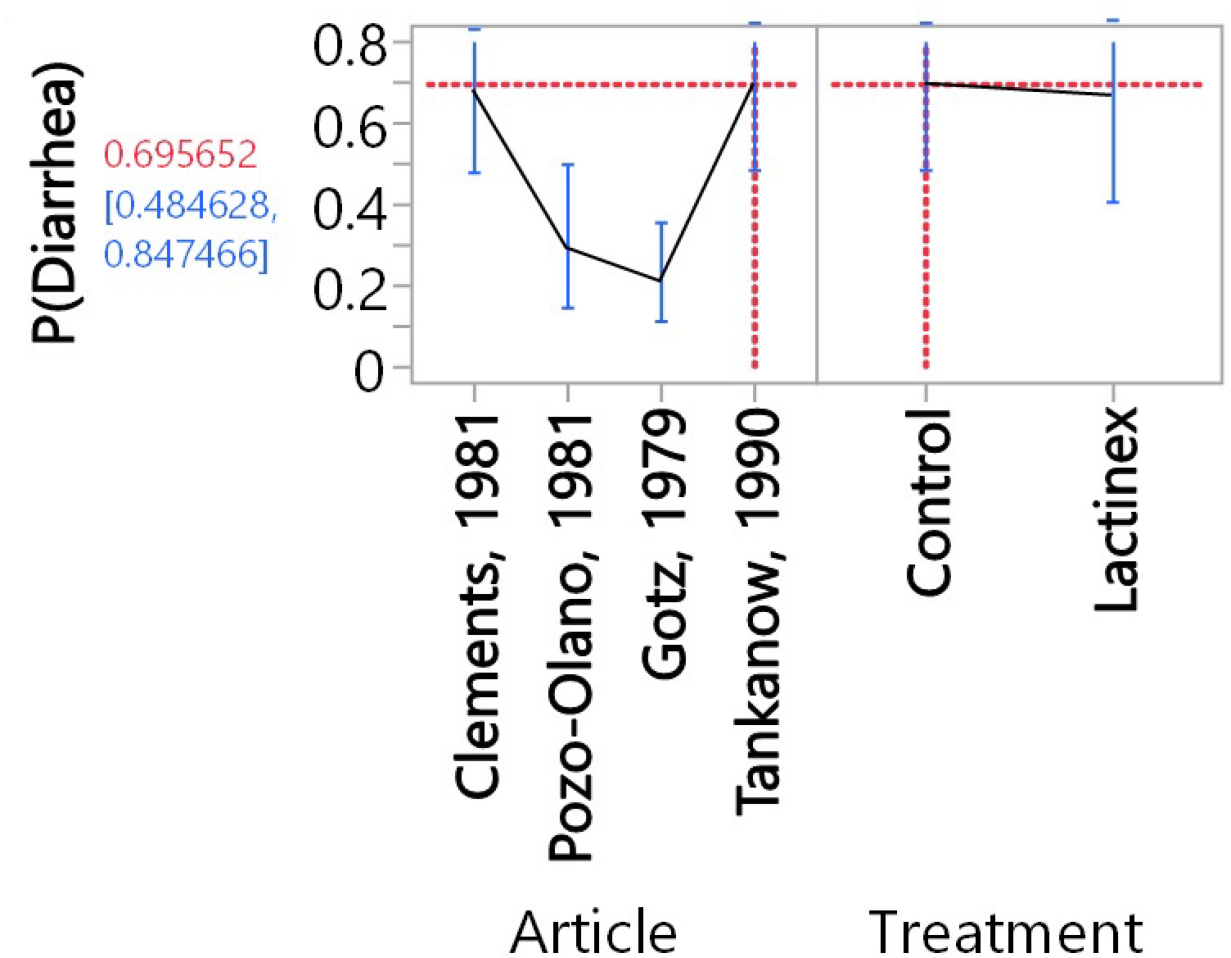
Prediction profiler resulting from the full-interaction generalized linear model. The vertical error bars represent the 95% confidence intervals, and the lines that are between these 95% confidence intervals connect mean values. The doted lines indicate that the highest proportion [69.6% (48.5%-84.8%, 95% confidence interval) of diarrhea was for Control in the Clements, 1981 article. The corresponding lowest proportion [8.3% (2.7%-22.9%)] of diarrhea was for Lactinex in the Gotz, 1979 article.

For the meta-analysis of probiotic treatment, there was also considerable heterogeneity (P < 0.0001), and this indicated that the proportion determined with random-effects meta-analysis should be used to interpret results. This proportion was 42.6% (14.3%─73.9, 95% confidence interval). Neither Begg’s test (P = 0.1742) nor Egger’s test (P = 0.1448) indicated publication bias. The generalized linear model analysis demonstrated differences (P < 0.0001), with the differences being mostly attributable to article (P < 0.0001) and not due to Treatment (P = 0.5081) nor to the interaction of Article and Treatment (P = 0.4941). The distributions of the studentized deviance residuals were judged to be acceptable and no cause for concern. The prediction profiler (**Figure 4**) created with the full interaction generalized linear model illustrates the large variation among articles compared with the small variation between treatments. In **Figure 4**, note that the mean values for placebo and probiotic are similar, and their 95% confidence intervals have considerable overlap, whereas for Articles, there is appreciable variation among the mean values, with some of the 95% confidence intervals not overlapping. These same relationships are present in **Figure 2**’s forest plots and **Figure 3** that contains the meta-analysis proportions for each study and the totals for random effects.

For some readers, it may be easier to compare placebo and probiotic with the proportions presented in **Figure 3**. To help readers assess the clinical importance of the difference in the overall proportions (as determined with the meta-analyses) for placebo and probiotic treatment, we performed a power analysis (sample-size calculation). Above, we present the rounded overall proportions for placebo and probiotic. The nonrounded values are 46.100% for placebo and 42.583% for probiotic. With alpha set at 0.05, a 2-tailed test (which means that an effect in either direction is interpreted), plus sample sizes of 4 for placebo and probiotic, an assessment of the difference in two independent proportions (46.100% versus 42.583%) would have a power of 5.1%. For a power of 80.0%, sample sizes of 3,133 articles would be required for placebo and for probiotic.

## 4 Discussion

To summarize the results of the meta-analyses, generalized linear model analysis, and sample-size calculation, the overall proportion of cases with diarrhea for the four probiotic articles was only 3.5% lower than the overall proportion for the four placebo articles (P = 0.5081), with our considering that the 3.5% lower percentage to be of little or no clinical importance (27). To demonstrate that this lower percentage would be statistically significant (at an alpha level of 0.05 with a power of 80.0%) would require 3,133 placebo and *L. acidophilus* and *L. bulgaricus* articles.

The outcome of diarrhea was limited by the included studies based on how it was defined in their methods. Because no singular definition of diarrhea exists between the included studies, the authors of this paper chose to use the definitions of diarrhea and outcome measurements as the original authors did. This allowed for a consistent outcome between studies allowing for a systematic review to be completed.

While a more specific outcome than diarrhea could not be defined due to the paucity of studies, the authors felt it proper to move forward with this review due to the widespread use of the investigated probiotic and the scarcity of scientific consensus surrounding the use of this probiotic.

All the available randomly controlled trials that studied the probiotic combination of *L. acidophilus* and *L. bulgaricus* were from before the turn of the century. Furthermore, all the available research articles that were found in this review failed to individually demonstrate a benefit to using *L. acidophilus and L. bulgaricus* for diarrhea, much less when looked at systematically.

It is, therefore, important to evaluate the worth of *L. acidophilus* and *L. bulgaricus* probiotic formulations when considering the nationwide push to value-based care (VBC) (28). While inexpensive, there is not enough available evidence to support continued usage of this formulation for diarrhea (28). At the very least, this warrants more current, large-scale studies to assess the efficacy of *L. acidophilus* and *L. bulgaricus* more accurately. However, in the absence of that data, *L. acidophilus* and *L. bugaricus* should be used judiciously, or not at all.

Finally, there are questions about the effectiveness of probiotics in general. There are studies that investigate *Lactobacillus* levels, and while there is a short-term spike in the concentration of the specific strain being supplemented, levels quickly return to baseline (29). Additionally, it is well established that gastric acid can interfere with probiotic colonization (30). This effect can be mediated by supplementation of PPIs to reduce gastric acid and increase colonization, but this introduces new variables and challenges to the microbiome that is already stressed (30). This brings into question whether there could be any benefit to probiotic supplementation if baseline is quickly restored with no changes in flora, and normal stomach secretions interfere with colonization downstream from the stomach. Any studies that look to the effectiveness of an *L. acidophilus* and *L. bulgaricus* formulation should also address the issues of colonization as there are numerous confounding variables at play.

### 4.1 Limitations

The largest limitation on this study is the lack of available evidence. Of the over two thousand articles reviewed, only 4 randomly controlled trials studied the combination of *L. acidophilus* and *L. bulgaricus* probiotic specifically for diarrhea, and none were in the last 10 years. Additionally, the causes of diarrhea and definitions of diarrhea were also diverse making direct comparison difficult. Of the studies that do exist, the n values were small and not able to be generalized to the population. Additionally, due to the quality of the available studies, it is not possible to assess the similarities of the populations in each study which further hinders generalization of these results.

## 5 Conclusions

Of the available research articles on *L. acidophilus* and *L. bulgaricus* probiotics as a treatment for diarrhea, there was not a statistical difference and little to no clinical difference between this treatment and placebo. While there were limitations to the study, there is not enough data to support its continued use in the treatment or prevention of all-cause diarrhea.

## Data availability statement

The original contributions presented in the study are included in the article/**Supplementary Material**. Further inquiries can be directed to the corresponding author.

## Author contributions

Study concept and design, acquisition of data, drafting of the manuscript: AC, DJ, and KR; statistical analysis and interpretation of data, critical revision of the manuscript for important intellectual content: CH; analysis and interpretation of data, critical revision of the manuscript for important intellectual content: WQ. All authors contributed to the article and approved the submitted version.

## Conflict of interest

The authors declare that the research was conducted in the absence of any commercial or financial relationships that could be construed as a potential conflict of interest.

## Publisher’s note

All claims expressed in this article are solely those of the authors and do not necessarily represent those of their affiliated organizations, or those of the publisher, the editors and the reviewers. Any product that may be evaluated in this article, or claim that may be made by its manufacturer, is not guaranteed or endorsed by the publisher.
