# Supplementary material for: A systematic review and meta-analysis of Lactobacillus acidophilus and Lactobacillus bulgaricus for the treatment of diarrhea
Source: Front Gastroenterol (Lausanne). 2022 Nov 9;1:983075. doi: 10.3389/fgstr.2022.983075 (PMC12952393; doi:10.3389/fgstr.2022.983075)
Supplement: Supplementary file 1 [file DataSheet_1.docx]

Details of statistical analyses

Based upon the systematic review, for the selected articles, the following data were collected from each article: (1) the number of placebo cases who developed diarrhea, (2) the total number of placebo cases, (3) the number of probiotic treated cases who developed diarrhea, (4) the total number of probiotic treated cases. With these data, two meta-analyses of the proportion of cases with diarrhea were performed—one meta-analysis for placebo and one meta-analysis for *L. acidophilus* and *L. bulgaricus* treatment. The proportion of cases with diarrhea was calculated as the number of cases with diarrhea divided by the total number of cases. A part of each meta-analysis was the calculation of weighted summary proportions (with fixed and random effects models) and the determination as to whether the fixed-effect or random-effects model should be used. With a fixed-effect model, it is assumed that the treatment effect is the same for all articles and that variation is due to sampling. With a random-effects model, treatment effects can vary among articles. Variation in treatment effects among articles is known as heterogeneity and was assessed with Cochran’s Q and I^2^ statistics. To determine the combined effect of the articles, weights are assigned to the different articles, with the weights being determined by the sample sizes and standard deviations of each article—that is, the inverse of the standard errors. Articles with larger sample sizes and smaller standard deviations are assigned higher weights in determining the combined effect. Assessments of publication bias were performed. Publication bias refers to the phenomenon that articles published in peer-refereed journals are much more likely to report statistically significant results than they are to report nonsignificant results, especially for smaller studies. Publication bias was assessed with Egger's test and Begg's test. To assist in interpreting meta-analysis results, a forest plot and funnel plot were created for each meta-analysis (Figure 1). Figure 2 was also created to make it easier for readers to assess the meta-analysis results for proportions of diarrhea for each study and the total random effects proportions for cplacebo and treatment.

An additional goal of our assessments was to determine whether there was a significant difference between cplacebo and probiotic groups for the overall proportions of cases who developed diarrhea, and for this assessment, we wanted to include the effect of the four articles. With general linear models, assumptions of normality are violated if the mean of the response is restricted to a specific range of values, such as a proportion (for which the range is 0% to 100%); however, this is not a problem with generalized linear models; therefore, to assess differences in proportions between placebo and probiotic, we used a generalized linear model with a binomial distribution and a logit link function. Because we wanted to assess how variation among the four articles could affect variation in proportions, for our predictor variables (x variables) of proportions (response variable/y variable), we included article (the four articles), treatment (cplacebo and probiotic), plus the interaction of article with treatment. For our generalized linear model analysis, plots of studentized deviance residuals were created and studied to detect trends that were not captured by the model. As part of the generalized linear model analysis, a prediction profiler was created to illustrate the variation among articles compared with the variation between treatments (Figure 3).

In a recently published article, it was recommended that the clinical importance of findings needs to be stressed, not merely their statistical significance [22]. The article was written by statisticians and scientists, had 800 signatories, plus many accompanying articles. With this recommendation in mind, we thought that rather than readers trying to assess the clinical importance of the difference in the overall proportions for cplacebo and probiotic treatment based upon our generalized linear model analysis, it would be relatively easy for them to assess the clinical importance of the difference between the overall proportions for cplacebo and for probiotic treatment that were calculated with meta-analyses. Because of this, we performed a power analysis (sample-size calculation) for testing the difference between two independent proportions (as determined with the meta-analyses) for cplacebo and probiotic treatment. For the analyses that were performed for our study, the alpha level was set at 0.05. Statistical analyses were performed with JMP Pro Statistical Software Release 16.2.0 (SAS Institute, Inc., Cary, NC), MedCalc Statistical Software version 20.104 (MedCalc Software Ltd, Ostend, Belgium; https://www.medcalc.org; 2022), and Power and Precision Release 4.1 (Biostat, Inc., Englewood, NJ).
